# The impact of methylation quantitative trait loci (mQTLs) on active smoking-related DNA methylation changes

**DOI:** 10.1186/s13148-017-0387-6

**Published:** 2017-08-17

**Authors:** Xu Gao, Hauke Thomsen, Yan Zhang, Lutz Philipp Breitling, Hermann Brenner

**Affiliations:** 1 Division of Clinical Epidemiology and Aging Research, German Cancer Research Center (DKFZ), Im Neuenheimer Feld 581, 69120 Heidelberg, Germany; 2 Division of Molecular Genetic Epidemiology, German Cancer Research Center (DKFZ), Im Neuenheimer Feld 580, 69120 Heidelberg, Germany; 3 Division of Preventive Oncology, German Cancer Research Center (DKFZ) and National Center for Tumor Diseases (NCT), Im Neuenheimer Feld 460, 69120 Heidelberg, Germany; 4 German Cancer Consortium (DKTK), German Cancer Research Center (DKFZ), Im Neuenheimer Feld 280, 69120 Heidelberg, Germany

**Keywords:** DNA methylation, Active smoking, Methylation quantitative trait loci, Epigenetic epidemiology

## Abstract

**Background:**

Methylation quantitative trait loci (mQTLs) are the genetic variants that may affect the DNA methylation patterns of CpG sites. However, their roles in influencing the disturbances of smoking-related epigenetic changes have not been well established. This study was conducted to address whether mQTLs exist in the vicinity of smoking-related CpG sites (± 50 kb) and to examine their associations with smoking exposure and all-cause mortality in older adults.

**Results:**

We obtained DNA methylation profiles in whole blood samples by Illumina Infinium Human Methylation 450 BeadChip array of two independent subsamples of the ESTHER study (discovery set, *n* = 581; validation set, *n* = 368) and their corresponding genotyping data using the Illumina Infinium OncoArray BeadChip. After correction for multiple testing (FDR), we successfully identified that 70 out of 151 previously reported smoking-related CpG sites were significantly associated with 192 SNPs within the 50 kb search window of each locus. The 192 mQTLs significantly influenced the active smoking-related DNA methylation changes, with percentage changes ranging from 0.01 to 18.96%, especially for the weakly/moderately smoking-related CpG sites. However, these identified mQTLs were not directly associated with active smoking exposure or all-cause mortality.

**Conclusions:**

Our findings clearly demonstrated that if not dealt with properly, the mQTLs might impair the power of epigenetic-based models of smoking exposure to a certain extent. In addition, such genetic variants could be the key factor to distinguish between the heritable and smoking-induced impact on epigenome disparities. These mQTLs are of special importance when DNA methylation markers measured by Illumina Infinium assay are used for any comparative population studies related to smoking-related cancers and chronic diseases.

**Electronic supplementary material:**

The online version of this article (doi:10.1186/s13148-017-0387-6) contains supplementary material, which is available to authorized users.

## Background

Active smoking has been recognized as a critical lifestyle factor for cardiovascular, respiratory, and neoplastic diseases and contributes to the leading causes of preventable morbidity and mortality [1, 2]. DNA methylation, one of the main forms of epigenetic modification, is involved in the pathways of smoking and smoking-induced diseases [3, 4]. Previous epigenome-wide association studies (EWASs) based on whole blood samples have successfully discovered an increasing number of tobacco smoking-related CpG sites in various genes, such as *AHRR* and *F2RL3* [5–7]. These DNA methylation patterns have been shown to be useful as quantitative biomarkers to reflect both current and lifetime smoking exposure and to enhance the prediction of smoking-related risks [8–11].

DNA methylation of particular genomic loci might be influenced by neighboring genetic sequence variants [12]. The single nucleotide polymorphisms (SNPs) that are associated with methylation levels of CpG sites are known as methylation quantitative trait loci (mQTLs) [13]. This genetic effect has been determined across different tissues [13–16] and has been highlighted in several diseases, including neurological disorders, arthritis, and cancer [17–21]. Recently, the mQTLs have been further reported to play a modifying role in the associations between DNA methylation levels at specific CpG sites and environmental exposures. For instance, Zhang et al. identified 238 mQTLs that were associated with 65 alcohol dependence-related CpG sites in African Americans and 305 mQTLs for 44 unique CpG sites in European Americans [22]. In 2016, Gonseth et al. found out that three of the strongest maternal smoking-related CpG sites in newborns were significantly associated with SNPs located in the vicinity of each gene [23]. Thus, these hereditary traits provide a possible mechanism by which methylation patterns could be different under environmental exposures, if the distribution of risk alleles differs between the exposed and the unexposed. In addition, the linkages of epigenetic signatures to genotypes might also further provide more mechanistic evidence on the genetic and environmental risk factors for various forms of diseases [24].

However, such genetic influences have not been well addressed or even overlooked by previous EWASs of active smoking exposure; to our knowledge, no study has so far investigated their contributions to the methylation intensities of active smoking-related CpG sites and smoking-related health outcomes in the general population. Therefore, we conducted a comprehensive analysis in a large population-based study of older adults in Germany with the aim of exploring the hitherto unknown association between active smoking-related DNA methylation and individual genetic variations. In particular, we aimed to identify the mQTLs within ± 50 kb from each of 151 previously reported active smoking-related CpG sites in whole blood samples [25] and to assess their relationships with active smoking exposure and all-cause mortality.

## Results

### Participant characteristics

Characteristics of the study population in the discovery and the validation panel with respect to smoking behaviors and lifestyle factors are summarized in Table 1. The average age of the participants of both subsets at the baseline was about 61 years. About half of the participants in each subset were ever smokers (current or former smokers), and around 18% still smoked at the time of recruitment. Female participants included a larger proportion of never smokers than males (discovery set, 67.9 vs. 28.6%; validation set, 63.3 vs. 21.4%). Average cumulative smoking exposure (pack-years) of current smokers was considerably higher than that of former smokers in both subsets (discovery set, 34.6 vs. 22.0; validation set, 33.1 vs. 19.4). Average time after smoking cessation (years) of former smokers in both subsets was also similar, approximately 17 years. The majority of participants in both subsets of the study population were overweight or obese, reported no or only low physical activity, and no or low amounts of alcohol drinking. During a median follow-up time of about 12 years (discovery set, 12.6 years; validation set, 12.2 years), 94 participants died in the discovery set (CVD = 30, cancer = 46, other diseases = 18) and 49 died in the validation set (CVD = 17, cancer = 21, other diseases = 11).

**Table 1:** Study population characteristics in discovery and validation panels (mean values (SD) for continuous variables and *n* (%) for categorical variables)

Characteristics	Discovery panel	Validation panel	*p* value
*N*	581	368	
Age (years)	61.0 (6.3)	61.1 (6.4)	0.809
Sex (male)	241 (41.5%)	117 (31.8%)	< 0.001
Smoking status			0.864
Current smoker	108 (18.6%)	65 (17.7%)	
Former smoker	173 (29.8%)	119 (32.3%)	
Never smoker	300 (51.6%)	184 (50.0%)	
Pack-years of smoking^a^			
Current smokers	34.6 (18.2)	33.1 (18.2)	0.250
Former smokers	22.0 (17.5)	19.4 (15.5)	0.033
Smoking cessation time (years)^b^	16.5 (11.3)	17.2 (10.2)	0.742
Body mass index^c^			0.248
Underweight or normal weight (< 25.0)	143 (24.7%)	116 (31.5%)	
Overweight (25.0–< 30.0)	290 (50.2%)	151 (41.0%)	
vObese (≥ 30.0)	145 (25.1%)	101 (27.5%)	
Alcohol consumption^d^			0.509
Abstainer	194 (36.3%)	128 (38.0%)	
Low	301 (56.4%)	188 (55.8%)	
Intermediate	30 (5.6%)	17 (5.0%)	
High	9 (1.7%)	4 (1.2%)	
Physical activity^e^			0.058
Inactive	109 (18.8%)	82 (22.3%)	
Low	245 (42.2%)	176 (47.8%)	
Medium or high	227 (39.0%)	110 (29.9%)	
Prevalence of CVD at baseline^f^			0.621
Prevalent	86 (14.8%)	58 (15.8%)	
Prevalence of diabetes at baseline^g^			0.617
Prevalent	86 (14.9%)	60 (16.6%)	
Prevalence of cancer at baseline			
Prevalent	33 (5.7%)	22 (6.0%)	0.744

^a^For subgroups of former and current smokers; data missing for 38 and 24 participants, respectively, in discovery and validation panels; a pack-year was defined as having smoked 20 cigarettes per day for 1 year

^b^Former smokers only, data missing for 5 and 2 participants, respectively, in discovery and validation panels; cessation time equals age at recruitment minus age at cessation

^c^Data missing for 3 participants in discovery panel

^d^Data missing for 47 and 31 participants, respectively, in discovery and validation panels. Categories defined as follows: abstainer, low [women, 0–< 20 g/d; men, 0–< 40 g/d], intermediate [20–< 40 g/d and 40–< 60 g/d, respectively], high [≥ 40 g/d and ≥ 60 g/d, respectively]

^e^Categories defined as follows: inactive [< 1 h of physical activity/week], medium or high [≥ 2 h of vigorous or ≥ 2 h of light physical activity/week], low (other)

^f^CVD cardiovascular disease. Data missing for 1 participant in discovery panel

^g^Data missing for 5 and 7 participants, respectively, in discovery and validation panels

### Identification of mQTLs for smoking-related CpG sites

**Fig. 1:**
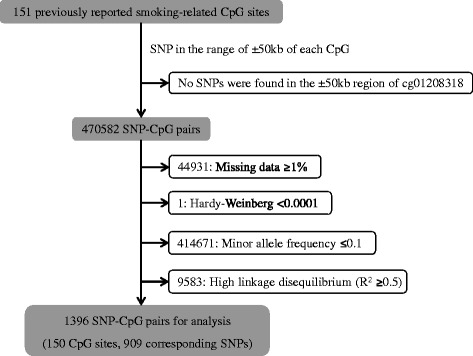
Flowchart of selection of SNP-CpG pairs

**Fig. 2:**
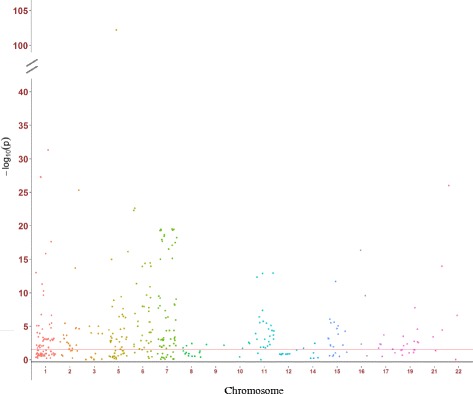
Manhattan plot of the results in validation panel. Red line, FDR-corrected *p* value = 0.05

**Fig. 3:**
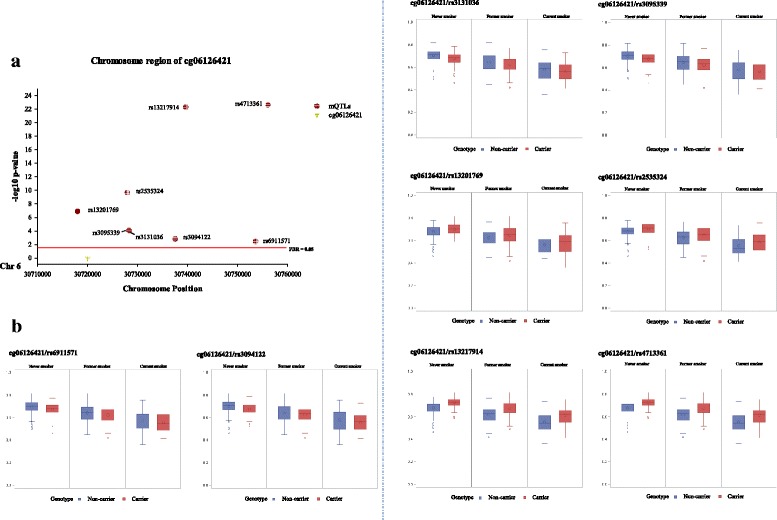
Locations of cg06126421 and eight mQTLs (carrier/non-carrier) (**a**) and distributions of methylation levels based on smoking status (**b**) in validation panel. Red line, FDR-corrected *p* value = 0.05; red dot, mQTLs; yellow triangle, cg06126421; blue bar, non-carriers of minor allele; red bar, carriers of minor allele

For the 1396 SNP-CpG pairs consisting of 150 smoking-related CpG sites and 909 corresponding SNPs (Fig. 1), 380 pairs were significant at a FDR < 0.05 in the discovery panel even after controlling for covariates (Additional file 1: Table S2; Additional file 2: Figure S1). These 380 pairs were then replicated in the validation panel by applying the fully adjusted mixed linear regression model. A subset of 246 pairs formed of 70 CpG sites and 192 SNPs reached the statistical significance level after FDR correction (FDR < 0.05; Table 2, Additional file 1: Table S3, Additional file 1: Table S4; Fig. 2). Eventually, 192 SNPs were designated as the mQTLs of 70 CpG sites. The pair cg23576855/rs75509302 showed the strongest inter-relationship (FDR-corrected *p* value = 8.86 e − 103). Among the 70 CpG sites, five were highly smoking-related loci (reported ≥ 6 times; Table 3), 14 were moderately smoking-related (4 or 5 times), and 51 were weakly smoking-related (2 or 3 times). These CpG sites with mQTLs were mainly located in the gene body (37/70), ten were located in transcription start sites (TSS1500) and 23 were in untranslated regions (UTR) or intergenetic regions (Additional file 1: Table S3). The largest number of mQTLs (*n* = 8) was found for locus cg06126421 within *6p21.33* (Table 3; Fig. 3). The coefficients of mQTLs ranged from −0.54 to 0.15.

**Table 2:** List of 246 significant SNP-CpG pairs (chromosomal and CpG sites positions were based on GRCh37/hg19)

Chromosome	Gene	CpG site	Position	Number of SNP candidates	Number of mQTLs
1	*AVPR1B*	cg09069072	15,482,754	13	5
	*GFI1*	cg09662411	92,946,132	10	2
		cg09935388	92,947,588	10	2
		cg10399789	92,945,668	9	6
		cg12876356	92,946,825	10	2
		cg18146737	92,946,701	10	2
		cg18316974	92,947,035	10	2
	*GNG12*	cg25189904	68,299,493	5	2
	*NOS1AP*	cg11231349	162,050,657	4	2
	*TMEM51*	cg21913886	15,485,346	14	9
	*unassigned*	cg03547355	227,003,061	8	2
	*unassigned*	cg12547807	9,473,751	9	1
	*unassigned*	cg21393163	12,217,630	8	2
	*unassigned*	cg26764244	68,299,511	3	3
2	*2q37.1*	cg05951221	233,284,402	5	1
		cg03329539	233,283,329	5	2
	*ALPP*	cg23667432	233,244,439	5	2
	*NFE2L2*	cg26271591	178,125,956	8	6
	*SNED1*	cg26718213	241,976,081	9	4
	*unassigned*	cg27241845	233,250,371	5	2
3	*GPX1*	cg18642234	49,394,623	10	5
5	*AHRR*	cg03604011	400,201	15	5
		cg03991871	368,448	9	1
		cg11902777	368,843	9	4
		cg12806681	368,395	9	2
		cg14817490	392,920	15	4
		cg17287155	393,347	15	1
		cg23576855	373,300	9	7
		cg23916896	368,805	9	4
6	*6p21.33*	cg06126421	30,720,081	16	8
	*CDKN1A*	cg15474579	36,645,813	22	8
	*IER3*	cg15342087	30,720,210	16	2
		cg24859433	30,720,204	16	3
	*TIAM2*	cg00931843	155,442,993	6	1
	*VARS*	cg17619755	31,760,629	16	8
	*unassigned*	cg14753356	30,720,109	8	2
7	*C7orf40*	cg03440944	45,023,330	5	1
	*CNTNAP2*	cg11207515	146,904,206	14	7
		cg25949550	145,814,306	11	8
	*GNA12*	cg19717773	2,847,554	22	22
	*HOXA7*	cg08396193	27,193,709	7	1
	*LRRN3*	cg11556164	110,738,316	5	5
	*MYO1G*	cg12803068	45,002,919	10	1
		cg22132788	45,002,487	10	1
	*TNRC18*	cg09022230	5,457,226	24	3
8	*ZC3H3*	cg26361535	144,576,604	8	1
	*unassigned*	cg19589396	103,937,374	15	2
9	*unassigned*	cg01692968	108,005,349	2	2
10	*ARID5B*	cg25953130	63,753,550	6	3
11	*KCNQ1*	cg07123182	2,722,391	13	2
		cg26963277	2,722,408	13	3
		cg01744331	2,722,358	13	3
	*KCNQ1OT1*	cg16556677	2,722,402	13	2
	*PRSS23*	cg11660018	86,510,915	9	2
		cg23771366	86,510,999	9	2
	*unassigned*	cg16611234	58,870,075	10	10
14	*C14orf43*	cg01731783	74,211,789	6	1
		cg10919522	74,227,441	5	1
15	*ANPEP*	cg23161492	90,357,203	19	6
	*SEMA7A*	cg00310412	74,724,919	13	9
16	*ITGAL*	cg09099830	30,485,486	3	2
	*XYLT1*	cg16794579	17,562,419	3	1
17	*LOC100130933*	cg07251887	73,641,810	6	2
	*STXBP4*	cg07465627	53,167,407	8	4
19	*CPAMD8*	cg15159987	17,003,890	15	4
	*CRTC1*	cg23973524	18,873,223	12	1
	*F2RL3*	cg03636183	17,000,586	17	1
	*PPP1R15A*	cg03707168	49,379,127	10	4
21	*ETS2*	cg23110422	40,182,073	6	3
22	*NCF4*	cg02532700	37,257,404	8	2
Total				590	246

**Table 3:** Five frequently reported (≥ 6) CpG sites and corresponding mQTLs

CpG site	Frequency^a^	Gene	Chr	SNP	SNP position^b^	Minor allele	Distance (bp)^c^	FDR^d^	MAF^e^
cg03636183	12	*F2RL3*	19	rs2227357	17,003,553	A	2967	0.048	0.125
cg05951221	8	*2q37.1*	2	rs790051	30,718,035	A	− 1866	6.2 e − 4	0.226
cg06126421	7	*6p21.33*	6	rs2535324	30,727,983	C	− 2046	1.8 e − 9	0.3
				rs3095339	30,728,290	G	7902	2.6 e − 4	0.252
				rs3131036	30,728,360	A	8209	2.6 e − 4	0.253
				rs3094122	30,737,552	G	8279	3.2 e − 3	0.206
				rs13217914	30,739,657	A	17,471	2.4 e − 21	0.157
				rs6911571	30,753,639	T	19,576	0.007	0.16
				rs4713361	30,756,066	A	33,558	1.3 e − 21	0.159
				rs13201769	30,718,035	A	35,985	6.9 e − 7	0.326
cg03329539	6	*2q37.1*	2	rs790051	233,282,536	A	− 793	0.031	0.226
				rs34547337	233,300,755	T	17,426	1.5 e − 5	0.314
cg14817490	6	*AHRR*	5	rs75509302	365,653	C	− 27,267	0.002	0.144
				rs11746079	410,980	C	18,060	1.5 e − 3	0.154
				rs72717419	431,996	T	39,076	0.021	0.207
				rs2672725	434,981	G	42,061	0.042	0.117

^a^The reported times of CpG in previous studies (based on systematic review [25])

^b^Positions of CpG sites and SNPs were based on GRCh37/hg19

^c^The distance between SNP and CpG (SNP position–CpG position)

^d^The FDR-corrected *p* values of SNPs in fully adjusted mixed linear regression models, which controlled for age (years), sex, smoking status, random batch effect of methylation measurement, leukocyte distribution (Houseman algorithm), alcohol consumption (abstainer/low/intermediate/high), body mass index (BMI, underweight or normal weight/overweight/obese), physical activity (inactive/low/medium or high), prevalence of cardiovascular diseases (yes/no), prevalence of diabetes (yes/no), and prevalence of cancer (yes/no)

*eMAF* minor allele frequency

The 192 mQTLs were mainly mapped on chromosomes 1 (16%), 6 (10%), and 7 (25%). Three SNPs were the most frequently identified mQTLs, rs75509392 (MAF = 0.144) for eight CpG sites within *AHRR*, rs79050605 (MAF = 0.202), and rs34835481 (MAF = 0.210) for five and six CpG sites located in *GFI1*, respectively (Table 4). We assessed the effects of the mQTLs on the DNA methylation changes as the absolute values of the coefficient changes of smoking status between the models without and with adjusting for corresponding mQTLs (carrier/non-carrier) among the 246 SNP-CpG pairs (Additional file 1: Table S5). Part of the mQTLs had opposite effects on different CpG sites (Additional file 1: Table S5, Additional file 3: Figure S2). For example, the minor allele of the SNP rs75509302 attenuated the association of smoking exposure with the methylation of cg11902777 by 5.2% (Additional file 1: Table S5, Additional file 3: Figure S2). In contrast, this variant strengthened the demethylation of cg17287155 in response to different smoking behaviors by 2.44%.

**Table 4:** Three most frequently identified mQTLs and corresponding CpG sites

SNP	Chr	SNP position^a^	Minor allele	MAF^b^	CpG	Distance (bp)^c^	FDR^d^
rs75509302	5	365,653	C	0.144	cg23576855	− 7647	3.4 e − 100
					cg11902777	− 3190	1.4 e − 7
					cg17287155	− 27,694	7.8 e − 5
					cg03991871	− 2795	1.0 e − 4
					cg12806681	− 2742	1.2 e − 4
					cg23916896	− 3152	9.5 e − 4
					cg03604011	− 34,548	1.1 e − 3
					cg14817490	− 27,267	2.1 e − 3
rs34835481	1	92,991,624	T	0.210	cg10399789	45,956	2.2 e − 5
					cg12876356	44,799	1.3 e − 3
					cg09662411	45,492	1.9 e − 3
					cg18146737	44,923	2.0 e − 3
					cg18316974	44,589	3.0 e − 3
					cg09935388	44,036	0.016
rs79050605	1	92,925,962	G	0.202	cg12876356	− 20,863	3.7 e − 4
					cg18146737	− 20,739	1.1 e − 3
					cg18316974	− 21,073	1.7 e − 3
					cg09662411	− 20,170	1.9 e − 3
					cg09935388	− 21,626	2.2 e − 3

^a^SNPs positions were based on GRCh37/hg19

^b^MAF minor allele frequency

^c^The distance between SNP and CpG (SNP position–CpG position)

^d^The FDR-corrected *p* values of SNPs in fully adjusted mixed linear regression models, which controlled for age (years), sex, smoking status, random batch effect of methylation measurement, leukocyte distribution (Houseman algorithm), alcohol consumption (abstainer/low/intermediate/high), body mass index (BMI, underweight or normal weight/overweight/obese), physical activity (inactive/low/medium or high), prevalence of cardiovascular diseases (yes/no), prevalence of diabetes (yes/no), and prevalence of cancer (yes/no)

### Genetic contributions of mQTLs to the DNA methylation changes

**Fig. 4:**
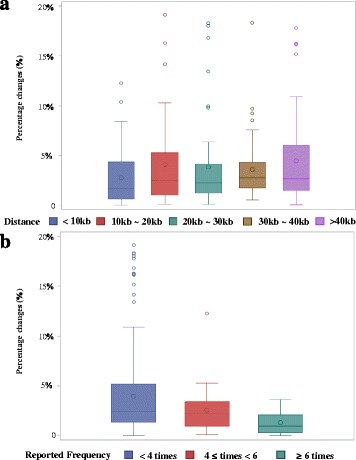
Percentage changes contributed by mQTLs to smoking-related DNA methylation changes based on SNP-CpG distance (**a**) and reported frequencies of CpG sites (**b**)

As shown in Additional file 1: Table S5, the associations between smoking exposure and DNA methylation were changed by between 0.01 and 18.96% by the mQTLs and were categorized by the distances between genetic variants and CpG sites and the reported frequencies of CpG sites. We observed that the closest SNPs (distance < 10 kb) had a slightly lower impact on DNA methylation levels than the mQTLs located ≥ 10 kb (Fig. 4a). Compared with the highly smoking-related CpG sites, the mQTLs affect the methylation levels of weakly smoking-related CpG sites the most, and the changes of moderately smoking-related CpG sites stayed in the intermediate position (Fig. 4b, *F* = 4.91, *p* value = 0.008). Additionally, potential gene-environment interactions of the 192 mQTLs with smoking behaviors (current/never smoking) were assessed. Only for rs75509302, a significant interaction with smoking was observed regarding the methylation levels of cg23576855 (Tables 5, Additional file 1: Table S6).

**Table 5:** Impact of rs75509302-smoking interaction on the methylation level of cg23576855^a^

Gene	CpG site	SNP	SNP-smoking interaction^b^				Smoking status^d^		
			Genotype^c^	Coefficient	SE	*p* value	Coefficient	SE	*p* value
*AHRR*	cg23576855	rs75509302	TT	Ref			−0.182	7.1 e − 3	3.8 e − 95
			CT	0.128	0.013	1.1 e − 20			
			CC	0.268	0.033	2.5 e − 15			

^a^Model is fully adjusted for age, sex, BMI, smoking status (current and never smoking only), alcohol consumption, physical activity, prevalence of CVD, diabetes and cancer at baseline. The methylation levels of CpG sites were responses, the SNPs and SNP-smoking interactions were predictors;

^b^The never smoking * genotype groups and current smoking * TT group were used as references;

^c^The group of interaction between current smoking and listed genotype;

^d^Never smoking was used as reference

### Associations of mQTLs with active smoking exposure and all-cause mortality

Finally, we tested the associations of the 192 mQTLs (carrier/non-carrier) with different measurements of smoking exposure [ever smoking (current and former smoking) vs. never smoking, current smoking vs. never smoking, current smoking vs. former smoking, cumulative smoking (pack-years), durations of smoking (years), and the age of smoking initiation]. None of them was significantly associated with any smoking indicators after the correction of FDR (Additional file 1: Table S7). Similarly, the 192 SNPs were not significantly associated with all-cause mortality (death from CVD, cancer, and other chronic diseases) based on the results of the COX model (Additional file 1: Table S8).

## Discussion

We conducted the first association study of 150 active smoking-related CpG sites and their corresponding SNPs located in the ± 50 kb region utilizing the genomic and epigenomic data of 949 participants from the ESTHER study. We found the DNA methylation levels of 70 CpG sites to be influenced by 192 proximal SNPs. These 192 mQTLs modified the DNA methylation changes in response to active smoking exposure, especially for the weakly/moderately active smoking-related CpG sites, but we did not observe any direct associations with active smoking exposure or all-cause mortality.

The mQTLs are presented in the vicinity of active smoking-related CpG sites. Locus cg06126421 (*6p21.33*) is one of the pronounced smoking-related CpG sites with eight mQTLs. Four of the SNPs could impair the hypomethylation of cg06126421, while others could accelerate this process. All of these mQTLs were located in a genomic region relating to inflammation and/or immune-related (malignant) diseases, such as allergies [26], multiple myeloma [27], diffuse large B cell lymphoma [28], and lung cancer [29]. We also identified a mQTL rs2227357 which could slightly promote the demethylation of cg03636183 (*F2RL3*), but no mQTLs were discovered near other well-established smoking-related loci, like cg05575921 (*AHRR*) or cg19859270 (*GPR15*). However, eight CpG sites within the *AHRR* region were found to be modified by mQTLs. Among them, cg23576855 manifested the strongest connection with rs75509302 and was also the only hit which could be affected by the interaction between smoking status and corresponding mQTL. This phenomenon might be highly contributed by the genetic feature of this locus, which is also a CG → CA SNP annotated as rs6869832 [5, 30], and shares a very high LD with rs75509302 in this study (R^2^ = 0.94). Nevertheless, to our knowledge, the biological functions for most of the identified mQTLs in our study are not fully understood yet and need to be explored in further research.

The mQTLs may help to distinguish between the genetic and environmental effects on epigenome disparities. Researchers usually observed several outliers out of the predictive range of epigenetic signatures in EWASs [6, 7, 9, 31]. One of the most plausible explanations is measurement bias that may result from recall bias or intentional underreporting [32]. Our finding provides another possibility that the deviations of DNA methylation levels might be caused by neighboring genetic variants. For highly smoking-related CpG sites, active smoking is still the main driver of DNA methylation changes. For instance, the SNP rs2227357 only contributed to about 0.01% of the changes of the methylation level of cg03636183 (*F2RL3*), and the SNP rs790051 altered only 0.37% of the methylation level of cg05951221 (*2q37.1*). However, the mQTLs affected the methylation levels of less robustly smoking-related loci much more. For instance, the SNPs rs78131 and rs2741302 explained nearly 19% of the changes of cg26963277 (*KCNQ1*) and cg27241845, respectively. While additional external validation studies certainly are needed, we speculate that this strong diversity of results might be a result of undetermined biological interactions between the SNP and CpG sites.

Smoking-related CpG sites have been recognized as informative signatures of smoking exposure and smoking-related health outcomes [3, 4]. Part of these 70 CpG sites with mQTLs have been reported to be highly associated with long-term smoking exposure [cg03636183 (*F2RL3*) and cg06126421 (*6p21.33*)] [9, 10], aging-related health outcomes, such as telomere length (cg21393163) [33] and the development of frailty [cg14753356, cg19589396, cg23667432 (*ALPP*) and cg25189904 (*GNG12*)] [34], and were even employed to construct a comprehensive index to predict smoking impact in buccal cells [35]. Together with previous studies [8, 23], we suggest that future investigations utilizing smoking-related CpG sites might need to take the genotypes, especially the mQTLs of less robustly smoking-related loci, into consideration to account for their potential impact on DNA methylation levels.

Beyond the SNP-CpG associations, null associations of 192 mQTLs with active smoking and all-cause mortality additionally imply that these novel genetic variants might be independently associated with the DNA methylation changes and might not be involved in the pathophysiological development of smoking-related health outcomes. Therefore, these mQTLs might have the potential to be used in the causal inference tests between the CpG sites and smoking-related health outcomes as instrumental variables (Mendelian Randomization, MR) [36]. Recently, researchers have suggested a two-stage MR test to establish the causal role of epigenetic processes in pathways of diseases [37]. Larger population-based investigations with longitudinal design and repeated measurements of smoking exposure and epigenome data are warranted to evaluate these potential instrumental variables and obtain further insights into the plausibility of suggested causal effects of DNA methylation in the development of smoking-related diseases.

Major strengths of the present study include comprehensive information on a broad range of covariates in a population-based cohort and validation in an independent subgroup. Some limitations still have to be acknowledged in the interpretation of study results. First, smoking-related shifts in leukocyte distribution might affect the associations of DNA methylation in whole blood samples with active smoking [38]. Hence, we adjusted for leukocyte distribution by the Houseman algorithm to restrict potential confounding from differential blood counts to the greatest possible extent [39]. Further studies are also needed to evaluate to what extent our results can be generalized to middle-aged individuals or non-Caucasians, as the ESTHER study was conducted in the older (aged 50–75 years), almost exclusively Caucasian population in southern Germany during a routine screening program. In addition, our study had limited power for detecting direct associations of mQTLs with smoking exposure and all-cause mortality due to limited numbers of cases. Finally, we only measured a relatively small window of genetic regions (± 50 kb) in whole blood DNA due to the consideration of controlling for the pleiotropic effect or reverse causations from unknown genetic or epigenetic factors and the limited coverage of OncoArray [40], more mQTLs (*cis*- or *trans*-) for smoking-related CpG sites need to be established by expanding the search window. Since DNA methylation is highly tissue-specific, larger cohorts with various human tissues are also needed for more comprehensive evaluation of the whole landscape of genetic impact on the epigenome.

## Conclusions

In conclusion, this study identified 192 mQTLs for 70 smoking-related CpG sites. These variants might theoretically reflect inherited differences in epigenetic states of people and their susceptibilities to smoking-related health outcomes. Incorporation of mQTLs might enhance the epigenetic-based assessments of smoking or smoking-related health outcomes by accounting for potential confounding from genetic background. Our results need to be further validated and confirmed in additional studies with larger number of participants and more detailed assessment of genomic and epigenomic data, including the CpG sites that have not previously been replicated. Along with previous investigations on the epigenetic changes related to other environmental exposures or lifestyle factors, our study adds evidence for the complex interplays among genetic traits, epigenetic signatures, and environmental factors.

## Methods

### Study design and population

Study subjects were selected from the ESTHER study, an ongoing statewide population-based cohort study conducted in Saarland, a state located in southwestern Germany. Details of the study design have been reported previously [41]. Briefly, 9949 older adults (aged 50–75 years) were enrolled by their general practitioners during a routine health check-up between July 2000 and December 2002 and followed up thereafter. The current cross-sectional analysis is based on data and biospecimen collected at baseline. Two independent subgroups were selected as the discovery and the validation panel for DNA methylation analyses as previously described [33]. Briefly, the discovery panel included 581 participants recruited consecutively at the start of the ESTHER study between July and October 2000. The validation panel included 368 participants randomly selected from the participants recruited between October 2000 and March 2001. The study was approved by the ethics committees of the University of Heidelberg and the state medical board of Saarland, Germany. Written informed consent was obtained from all participants.

### Data collection

Information on socio-demographic characteristics, lifestyle factors, and health status at baseline was obtained by standardized self-administered questionnaires. In particular, detailed information on lifetime smoking history was obtained, including current smoking status and intensity, age at initiation, and smoking intensities at various ages, as well as the age of quitting smoking for former smokers [42]. Additional information on body mass index (BMI) was extracted from a standardized form filled by the general practitioners during the health check-ups. Prevalent cardiovascular disease (CVD) at baseline was defined by either physician-reported coronary heart disease or a self-reported history of a major cardiovascular event, such as myocardial infarction, stroke, pulmonary embolism, or revascularization of coronary arteries. Prevalent diabetes was defined by physician diagnosis or the use of glucose-lowering drugs. Prevalent cancer [ICD-10 C00-C99 except non-melanoma skin cancer (C44)] was determined by self-report or record linkage with data from the Saarland Cancer Registry (http://www.krebsregister.saarland.de/ziele/ziel1.html; in German). Deaths during the follow-up (between 2000 and end of 2014) were identified by record linkage with population registries in Saarland. Participants migrated out of Saarland were censored at the date last known to be alive. Information about the major cause of death was obtained from death certificates provided by the local public health offices and was coded with ICD-10 codes.

### DNA methylation data

Blood samples were taken during the health check-up and stored at −80 °C until further processing. DNA from whole blood samples was collected using a salting out procedure [43]. DNA methylation profiles were extracted by the Illumina Human Methylation 450K BeadChip (Illumina, San Diego, CA, USA). As previously described [44], samples were analyzed following the manufacturer’s instruction at the Genomics and Proteomics Core Facility of the German Cancer Research Center, Heidelberg, Germany. Illumina’s GenomeStudio® (version 2011.1; Illumina, Inc.) was employed to extract DNA methylation signals from the scanned arrays (Module version 1.9.0; Illumina, Inc.). The methylation level of a specific CpG site was quantified as a *β* value ranging from 0 (no methylation) to 1 (full methylation). According to the manufacturer’s protocol, no background correction was done and data were normalized to internal controls provided by the manufacturer. All controls were checked for inconsistencies in each measured plate. Probes with a detection *p* value > 0.05 were excluded from analysis. We utilized the Illumina normalization and preprocessing method implemented in Illumina’s GenomeStudio®. We selected the profiles of 151 smoking-related loci which had been identified ≥ 2 times in previous smoking EWASs for the present analysis [25].

### Genotyping data

Extracted DNA from blood cells was genotyped using the Illumina Infinium OncoArray BeadChip (Illumina, San Diego, CA, USA). General genotyping quality control assessment was as previously described [45]. Genotypes for common variants across the genome were imputed using data from 1000 Genomes Project (phase 3, Oct. 2014) with IMPUTE2 v2.3.2 after pre-phasing with SHAPEIT software v2.12. We set thresholds for imputation quality to retain both potential common and rare variants for validation. Specifically, poorly imputed SNPs defined by an information metric *I* < 0.70 were excluded. All genomic locations are given in NCBI Build 37/UCSC hg19 coordinates. All SNPs having a MAF < 1% were excluded. After imputation, the SNP set consisted of 9,198,808 genotyped and imputed SNPs. PLINK v1.90 was then used to extract SNPs for the required regions of interest [46]. As shown in Fig. 1, we first identified SNPs within 50 kb upstream and downstream from each of the 151 smoking-related CpG sites (470,582 SNP-CpG pairs), a window in which most SNPs with significant *cis* associations with CpG sites are located [13]. The locus cg01208318 was excluded without any corresponding SNPs in this restricted region. For each of the remaining 150 CpG sites, we excluded any SNPs with ≥ 1% missing values (*n* = 44,931), deviating from the Hardy-Weinberg equilibrium (HWE exact test’s *p* value < 0.0001, *n* = 1), with a minor allele frequency ≤ 0.1 (*n* = 414,671) or with high linkage disequilibrium (LD, *R* ^2^ ≥ 0.5) (Additional file 1: Table S1). After the final quality control, 1396 SNP-CpG pairs with strongest SNPs remained for analysis, which were constituted of 150 CpG sites and 909 corresponding SNPs (Additional file 1: Table S2).

### Statistical analyses

First, major socio-demographic characteristics, lifestyle factors, smoking behavior, and prevalence of major chronic diseases in both the discovery and the validation panel were summarized by descriptive statistics.

We then evaluated the associations between the methylation intensities of the 150 CpG sites and corresponding SNPs to identify mQTLs as follows. For all SNP-CpG pairs, we used a mixed linear regression model with methylation batch as a random effect in which the methylation level of CpG site was the outcome and each regional SNP was the predictor (categorical variable, coded into 0, 1, and 2 based on the numbers of the minor allele). The model was fully adjusted for the following covariates that have been shown to be associated with DNA methylation changes [47–54]: age (years), sex (male/female), smoking status (current/former/never smoking), alcohol consumption (abstainer, low [women, 0 to < 20 g/d; men, 0 to < 40 g/d], intermediate [20 to < 40 g/d and 40 to < 60 g/d, respectively], high [≥ 40 g/d and ≥ 60 g/d, respectively]), body mass index (BMI, kg/m^2^, underweight [< 18.5, < 1% of the study population] or normal weight [18.5 to < 25], overweight [25 to < 30], obese [≥ 30]), physical activity (inactive [< 1 h of physical activity/week], medium or high [≥ 2 h of vigorous or ≥ 2 h of light physical activity/week], low [other]), the leukocyte distribution estimated by the Houseman algorithm [39], the prevalence of CVD (yes/no), diabetes (yes/no), and cancer (yes/no) at the baseline. After correction for multiple testing by false discovery rate (FDR, Benjamini-Hochberg method [55]), SNP-CpG pairs with a FDR < 0.05 were selected and then analyzed in the validation panel. SNPs of the pairs with a FDR < 0.05 in the validation panel were eventually identified as the mQTL for the corresponding CpG site.

Furthermore, we tested the contributions of the identified mQTLs to the DNA methylation levels of corresponding CpG sites. Due to the limited number of individuals in the subgroup of minor homozygotes, we recoded the SNPs in order to use the dominant model, in which the heterozygote and minor homozygotes were combined as the carrier of minor allele and the major homozygotes were considered as non-carrier of the minor allele. We compared the coefficients of active smoking exposure (current vs. never smoking) in the fully adjusted model without the mQTLs (*β*_1_) with the fully adjusted model including the mQTLs (*β*_2_). The changes of coefficients were calculated as 100% * (*β*_1_–β_2_)/*β*_1_, and their absolute values were determined as the percentage change contributed by mQTLs. The percentage changes were categorized by the absolute distances (bp) between CpG sites and corresponding mQTLs and the reported frequencies of CpG sites. To explore whether the gene-environment interactions could modify the DNA methylation changes of smoking-related CpG sites, we also tested whether interactions between the identified mQTLs and active smoking exposure (current vs. never smoking) could affect the impact of smoking on the methylation levels of corresponding CpG sites. The mQTLs, smoking status, and their interaction (mQTLs*smoking status) were added in the model as predictors, and the methylation levels of CpG sites were outcomes. After controlling for all the potential covariates, the interactions with a FDR < 0.05 were considered as methylation-related interactions for corresponding CpG sites.

Finally, we examined whether the identified mQTLs (carrier/non-carrier) were associated with six active smoking indicators: ever smoking (current and former smoking) vs. never smoking, current smoking vs. never smoking, current smoking vs. former smoking, cumulative smoking (pack-years), durations of smoking (years), and the age of smoking initiation. The mixed linear models were fully adjusted for age (years), sex, smoking status, alcohol consumption, BMI, physical activity, the prevalence of CVD, diabetes, and cancer as described above. The mQTLs with a FDR < 0.05 were identified as smoking-related SNPs. We also assessed the associations of the significant mQTLs (carrier/non-carrier) with all-cause mortality in ESTHER study. Due to the limited number of deaths, we combined both subsets and performed the analysis using a multiple COX regression model. The model was adjusted for the above potential covariates and SNPs with a FDR < 0.05 were considered as all-cause mortality related variants.

Data cleaning and all aforementioned statistical analyses were performed by SAS version 9.4 (SAS Institute Inc., Cary, NC, USA). Manhattan plots for both panels were plotted by R package “ggplot2.”

## Additional files

Additional file 1: Table S1. Basic information of genomic data for 151 smoking-related CpG sites. **Table S2** List of 1396 significant SNP-CpG pairs in discovery panel. **Table S3** List of 246 significant SNP-CpG pairs in validation panel. **Table S4** Results of regression analyses for 246 SNP-CpG pairs in validation panel. **Table S5** Estimate changes of smoking status (never/current smoking) between models with and without adjusting for mQTLs. **Table S6** Impacts of mQTL-smoking interaction on the methylation levels of corresponding CpG sites in 949 participants (never/current smoking). **Table S7** Associations of 192 mQTLs with active smoking indicators. **Table S8.** Associations of 192 mQTLs with all-cause mortality. (XLSX 263 kb)

Additional file 2: Figure S1. Manhattan plot of the results in discovery panel. (PDF 85 kb)

Additional file 3: Figure S2. Locations and distributions of methylation levels of 19 smoking-related CpG sites based on the three most frequently identified mQTLs (carrier/non-carrier) and smoking status in validation panel. (PDF 206 kb)

## Abbreviations

CpG sites: Cytosine-phosphate-guanine sites
CVD: Cardiovascular disease
EWASs: Epigenome-wide association studies
FDR: False discovery rate
mQTLs: Methylation quantitative trait loci
SNPs: Single nucleotide polymorphisms

## References

[1] Babizhayev MA, Yegorov YE (2011). Smoking and health: association between telomere length and factors impacting on human disease, quality of life and life span in a large population-based cohort under the effect of smoking duration. Fundam Clin Pharmacol.

[2] Mathers CD, Loncar D (2006). Projections of global mortality and burden of disease from 2002 to 2030. PLoS Med.

[3] Philibert RA, Beach SR, Brody GH (2014). The DNA methylation signature of smoking: an archetype for the identification of biomarkers for behavioral illness. Neb Symp Motiv.

[4] Lee KW, Pausova Z (2013). Cigarette smoking and DNA methylation. Front Genet.

[5] Philibert RA, Beach SR, Lei MK, Brody GH (2013). Changes in DNA methylation at the aryl hydrocarbon receptor repressor may be a new biomarker for smoking. Clin Epigenetics.

[6] Zeilinger S, Kuhnel B, Klopp N, Baurecht H, Kleinschmidt A, Gieger C, Weidinger S, Lattka E, Adamski J, Peters A (2013). Tobacco smoking leads to extensive genome-wide changes in DNA methylation. PLoS One.

[7] Breitling LP, Yang R, Korn B, Burwinkel B, Brenner H (2011). Tobacco-smoking-related differential DNA methylation: 27K discovery and replication. Am J Hum Genet.

[8] Qiu W, Wan E, Morrow J, Cho MH, Crapo JD, Silverman EK, DeMeo DL (2015). The impact of genetic variation and cigarette smoke on DNA methylation in current and former smokers from the COPDGene study. Epigenetics.

[9] Shenker NS, Ueland PM, Polidoro S, van Veldhoven K, Ricceri F, Brown R, Flanagan JM, Vineis P (2013). DNA methylation as a long-term biomarker of exposure to tobacco smoke. Epidemiology.

[10] Zhang Y, Yang R, Burwinkel B, Breitling LP, Brenner H (2014). F2RL3 methylation as a biomarker of current and lifetime smoking exposures. Environ Health Perspect.

[11] Tsaprouni LG, Yang TP, Bell J, Dick KJ, Kanoni S, Nisbet J, Vinuela A, Grundberg E, Nelson CP, Meduri E (2014). Cigarette smoking reduces DNA methylation levels at multiple genomic loci but the effect is partially reversible upon cessation. Epigenetics.

[12] Gutierrez-Arcelus M, Lappalainen T, Montgomery SB, Buil A, Ongen H, Yurovsky A, Bryois J, Giger T, Romano L, Planchon A (2013). Passive and active DNA methylation and the interplay with genetic variation in gene regulation. elife.

[13] Bell JT, Pai AA, Pickrell JK, Gaffney DJ, Pique-Regi R, Degner JF, Gilad Y, Pritchard JK (2011). DNA methylation patterns associate with genetic and gene expression variation in HapMap cell lines. Genome Biol.

[14] Gibbs JR, van der Brug MP, Hernandez DG, Traynor BJ, Nalls MA, Lai SL, Arepalli S, Dillman A, Rafferty IP, Troncoso J (2010). Abundant quantitative trait loci exist for DNA methylation and gene expression in human brain. PLoS Genet.

[15] Schalkwyk LC, Meaburn EL, Smith R, Dempster EL, Jeffries AR, Davies MN, Plomin R, Mill J (2010). Allelic skewing of DNA methylation is widespread across the genome. Am J Hum Genet.

[16] Kerkel K, Spadola A, Yuan E, Kosek J, Jiang L, Hod E, Li K, Murty VV, Schupf N, Vilain E (2008). Genomic surveys by methylation-sensitive SNP analysis identify sequence-dependent allele-specific DNA methylation. Nat Genet.

[17] Gamazon ER, Badner JA, Cheng L, Zhang C, Zhang D, Cox NJ, Gershon ES, Kelsoe JR, Greenwood TA, Nievergelt CM (2013). Enrichment of cis-regulatory gene expression SNPs and methylation quantitative trait loci among bipolar disorder susceptibility variants. Mol Psychiatry.

[18] Rushton MD, Reynard LN, Young DA, Shepherd C, Aubourg G, Gee F, Darlay R, Deehan D, Cordell HJ, Loughlin J (2015). Methylation quantitative trait locus analysis of osteoarthritis links epigenetics with genetic risk. Hum Mol Genet.

[19] Heyn H, Sayols S, Moutinho C, Vidal E, Sanchez-Mut JV, Stefansson OA, Nadal E, Moran S, Eyfjord JE, Gonzalez-Suarez E (2014). Linkage of DNA methylation quantitative trait loci to human cancer risk. Cell Rep.

[20] Liu Y, Aryee MJ, Padyukov L, Fallin MD, Hesselberg E, Runarsson A, Reinius L, Acevedo N, Taub M, Ronninger M (2013). Epigenome-wide association data implicate DNA methylation as an intermediary of genetic risk in rheumatoid arthritis. Nat Biotechnol.

[21] Li Q, Seo JH, Stranger B, McKenna A, Pe'er I, Laframboise T, Brown M, Tyekucheva S, Freedman ML (2013). Integrative eQTL-based analyses reveal the biology of breast cancer risk loci. Cell.

[22] Zhang H, Wang F, Kranzler HR, Yang C, Xu H, Wang Z, Zhao H, Gelernter J (2014). Identification of methylation quantitative trait loci (mQTLs) influencing promoter DNA methylation of alcohol dependence risk genes. Hum Genet.

[23] Gonseth S, de Smith AJ, Roy R, Zhou M, Lee ST, Shao X, Ohja J, Wrensch MR, Walsh KM, Metayer C, Wiemels JL. Genetic contribution to variation in DNA methylation at maternal smoking sensitive loci in exposed neonates. Epigenetics. 2016:0. 10.1080/15592294.2016.1209614 PMC5048731 27403598

[24] Ladd-Acosta C, Fallin MD (2016). The role of epigenetics in genetic and environmental epidemiology. Epigenomics.

[25] Gao X, Jia M, Zhang Y, Breitling LP, Brenner H (2015). DNA methylation changes of whole blood cells in response to active smoking exposure in adults: a systematic review of DNA methylation studies. Clin Epigenetics.

[26] Hinds DA, McMahon G, Kiefer AK, Do CB, Eriksson N, Evans DM, St Pourcain B, Ring SM, Mountain JL, Francke U (2013). A genome-wide association meta-analysis of self-reported allergy identifies shared and allergy-specific susceptibility loci. Nat Genet.

[27] Chubb D, Weinhold N, Broderick P, Chen B, Johnson DC, Forsti A, Vijayakrishnan J, Migliorini G, Dobbins SE, Holroyd A (2013). Common variation at 3q26.2, 6p21.33, 17p11.2 and 22q13.1 influences multiple myeloma risk. Nat Genet.

[28] Cerhan JR, Berndt SI, Vijai J, Ghesquieres H, McKay J, Wang SS, Wang Z, Yeager M, Conde L, de Bakker PI (2014). Genome-wide association study identifies multiple susceptibility loci for diffuse large B cell lymphoma. Nat Genet.

[29] Jin G, Zhu M, Yin R, Shen W, Liu J, Sun J, Wang C, Dai J, Ma H, Wu C (2015). Low-frequency coding variants at 6p21.33 and 20q11.21 are associated with lung cancer risk in Chinese populations. Am J Hum Genet.

[30] Shenker NS, Polidoro S, van Veldhoven K, Sacerdote C, Ricceri F, Birrell MA, Belvisi MG, Brown R, Vineis P, Flanagan JM (2013). Epigenome-wide association study in the European Prospective Investigation into Cancer and Nutrition (EPIC-Turin) identifies novel genetic loci associated with smoking. Hum Mol Genet.

[31] Zhang Y, Florath I, Saum KU, Brenner H (2016). Self-reported smoking, serum cotinine, and blood DNA methylation. Environ Res.

[32] Connor Gorber S, Schofield-Hurwitz S, Hardt J, Levasseur G, Tremblay M (2009). The accuracy of self-reported smoking: a systematic review of the relationship between self-reported and cotinine-assessed smoking status. Nicotine Tob Res.

[33] Gao X, Mons U, Zhang Y, Breitling LP, Brenner H (2016). DNA methylation changes in response to active smoking exposure are associated with leukocyte telomere length among older adults. Eur J Epidemiol.

[34] Gao X, Zhang Y, Saum KU, Schottker B, Breitling LP, Brenner H (2017). Tobacco smoking and smoking-related DNA methylation are associated with the development of frailty among older adults. Epigenetics.

[35] Teschendorff AE, Yang Z, Wong A, Pipinikas CP, Jiao Y, Jones A, Anjum S, Hardy R, Salvesen HB, Thirlwell C (2015). Correlation of smoking-associated DNA methylation changes in buccal cells with DNA methylation changes in epithelial cancer. JAMA Oncol.

[36] Davey Smith G, Hemani G. Mendelian randomization: genetic anchors for causal inference in epidemiological studies. Hum Mol Genet. 2014;23:R89–98. 10.1093/hmg/ddu328 PMC4170722 25064373

[37] Relton CL, Davey Smith G (2012). Two-step epigenetic Mendelian randomization: a strategy for establishing the causal role of epigenetic processes in pathways to disease. Int J Epidemiol.

[38] Schwartz J, Weiss ST (1994). Cigarette smoking and peripheral blood leukocyte differentials. Ann Epidemiol.

[39] Houseman EA, Accomando WP, Koestler DC, Christensen BC, Marsit CJ, Nelson HH, Wiencke JK, Kelsey KT (2012). DNA methylation arrays as surrogate measures of cell mixture distribution. BMC Bioinformatics.

[40] Lande R (1980). The genetic covariance between characters maintained by pleiotropic mutations. Genetics.

[41] Schöttker B, Haug U, Schomburg L, Kohrle J, Perna L, Muller H, Holleczek B, Brenner H (2013). Strong associations of 25-hydroxyvitamin D concentrations with all-cause, cardiovascular, cancer, and respiratory disease mortality in a large cohort study. Am J Clin Nutr.

[42] Gao X, Gao X, Zhang Y, Breitling LP, Schottker B, Brenner H (2017). Associations of self-reported smoking, cotinine levels and epigenetic smoking indicators with oxidative stress among older adults: a population-based study. Eur J Epidemiol.

[43] Miller SA, Dykes DD, Polesky HF (1988). A simple salting out procedure for extracting DNA from human nucleated cells. Nucleic Acids Res.

[44] Florath I, Butterbach K, Heiss J, Bewerunge-Hudler M, Zhang Y, Schöttker B, Brenner H (2015). Type 2 diabetes and leucocyte DNA methylation: an epigenome-wide association study in over 1,500 older adults. Diabetologia.

[45] Anderson CA, Pettersson FH, Clarke GM, Cardon LR, Morris AP, Zondervan KT (2010). Data quality control in genetic case-control association studies. Nat Protoc.

[46] Chang CC, Chow CC, Tellier LC, Vattikuti S, Purcell SM, Lee JJ (2015). Second-generation PLINK: rising to the challenge of larger and richer datasets. Gigascience.

[47] Shen-Orr SS, Tibshirani R, Khatri P, Bodian DL, Staedtler F, Perry NM, Hastie T, Sarwal MM, Davis MM, Butte AJ (2010). Cell type-specific gene expression differences in complex tissues. Nat Methods.

[48] Philibert RA, Plume JM, Gibbons FX, Brody GH, Beach SR (2012). The impact of recent alcohol use on genome wide DNA methylation signatures. Front Genet.

[49] Jones MJ, Goodman SJ, Kobor MS (2015). DNA methylation and healthy human aging. Aging Cell.

[50] Dick KJ, Nelson CP, Tsaprouni L, Sandling JK, Aissi D, Wahl S, Meduri E, Morange PE, Gagnon F, Grallert H (2014). DNA methylation and body-mass index: a genome-wide analysis. Lancet.

[51] Zhang FF, Cardarelli R, Carroll J, Zhang S, Fulda KG, Gonzalez K, Vishwanatha JK, Morabia A, Santella RM (2011). Physical activity and global genomic DNA methylation in a cancer-free population. Epigenetics.

[52] Nilsson E, Jansson PA, Perfilyev A, Volkov P, Pedersen M, Svensson MK, Poulsen P, Ribel-Madsen R, Pedersen NL, Almgren P (2014). Altered DNA methylation and differential expression of genes influencing metabolism and inflammation in adipose tissue from subjects with type 2 diabetes. Diabetes.

[53] Breitling LP (2013). Current genetics and epigenetics of smoking/tobacco-related cardiovascular disease. Atertio Thromb Vasc Biol.

[54] Shen H, Laird PW (2013). Interplay between the cancer genome and epigenome. Cell.

[55] Benjamini Y, Hochberg Y (1995). Controlling the false discovery rate––a practical and powerful approach to multiple testing. J R Stat Soc Series B Stat Methodol.

