# Supplementary figures and images for: The impact of methylation quantitative trait loci (mQTLs) on active smoking-related DNA methylation changes
Source: Clin Epigenetics. 2017 Aug 17;9:87. doi: 10.1186/s13148-017-0387-6 (PMC5561570; doi:10.1186/s13148-017-0387-6)

**Figure S1** Manhattan plot of the results in discovery panel

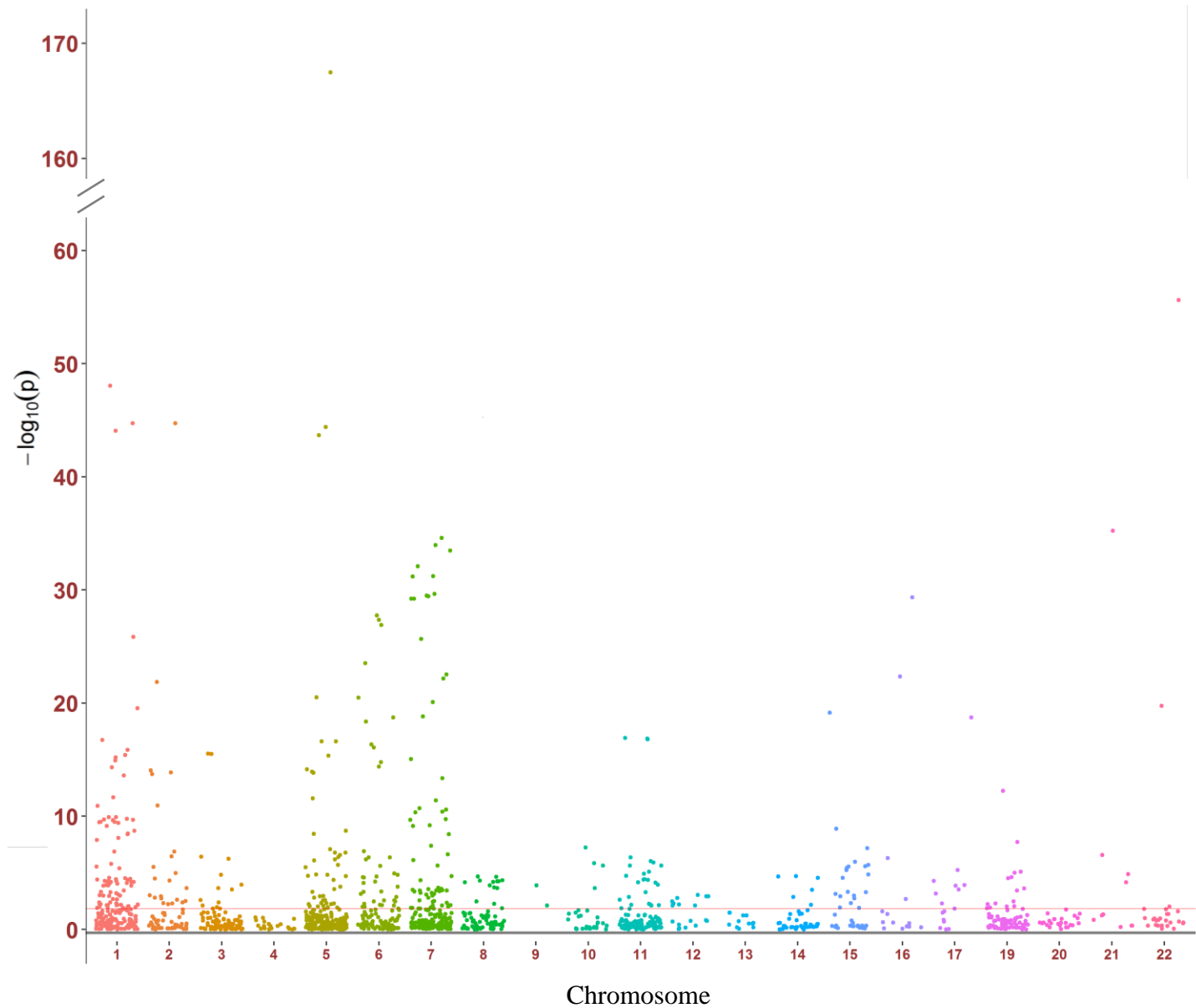

Supplement: Supplementary file 2 — Manhattan plot of the results in discovery panel. (PDF 85 kb) [file 13148_2017_387_MOESM2_ESM.pdf]
