# Supplementary material for: The impact of methylation quantitative trait loci (mQTLs) on active smoking-related DNA methylation changes
Source: Clin Epigenetics. 2017 Aug 17;9:87. doi: 10.1186/s13148-017-0387-6 (PMC5561570; doi:10.1186/s13148-017-0387-6)
Supplement: Supplementary file 3 — Locations and distributions of methylation levels of 19 smoking-related CpG sites based on the three most frequently identified mQTLs (carrier/non-carrier) and smoking status in validation panel. (PDF 206 kb) [file 13148_2017_387_MOESM3_ESM.pdf]

Figure S2 Locations and distributions of methylation levels of 19 smoking-related CpG sites based on the three most frequently identified mQTLs (carrier/non-carrier) and smoking status in validation panel

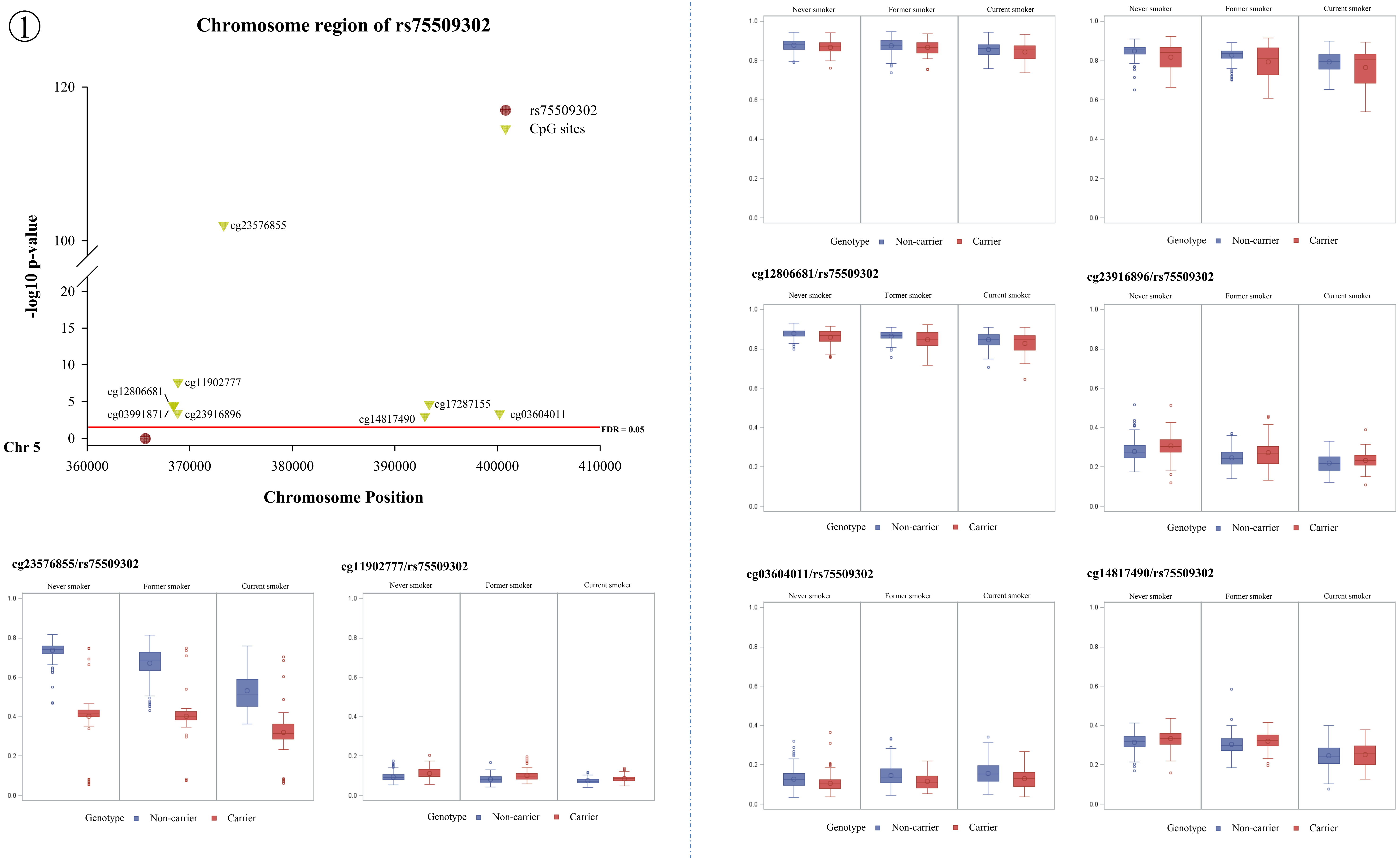

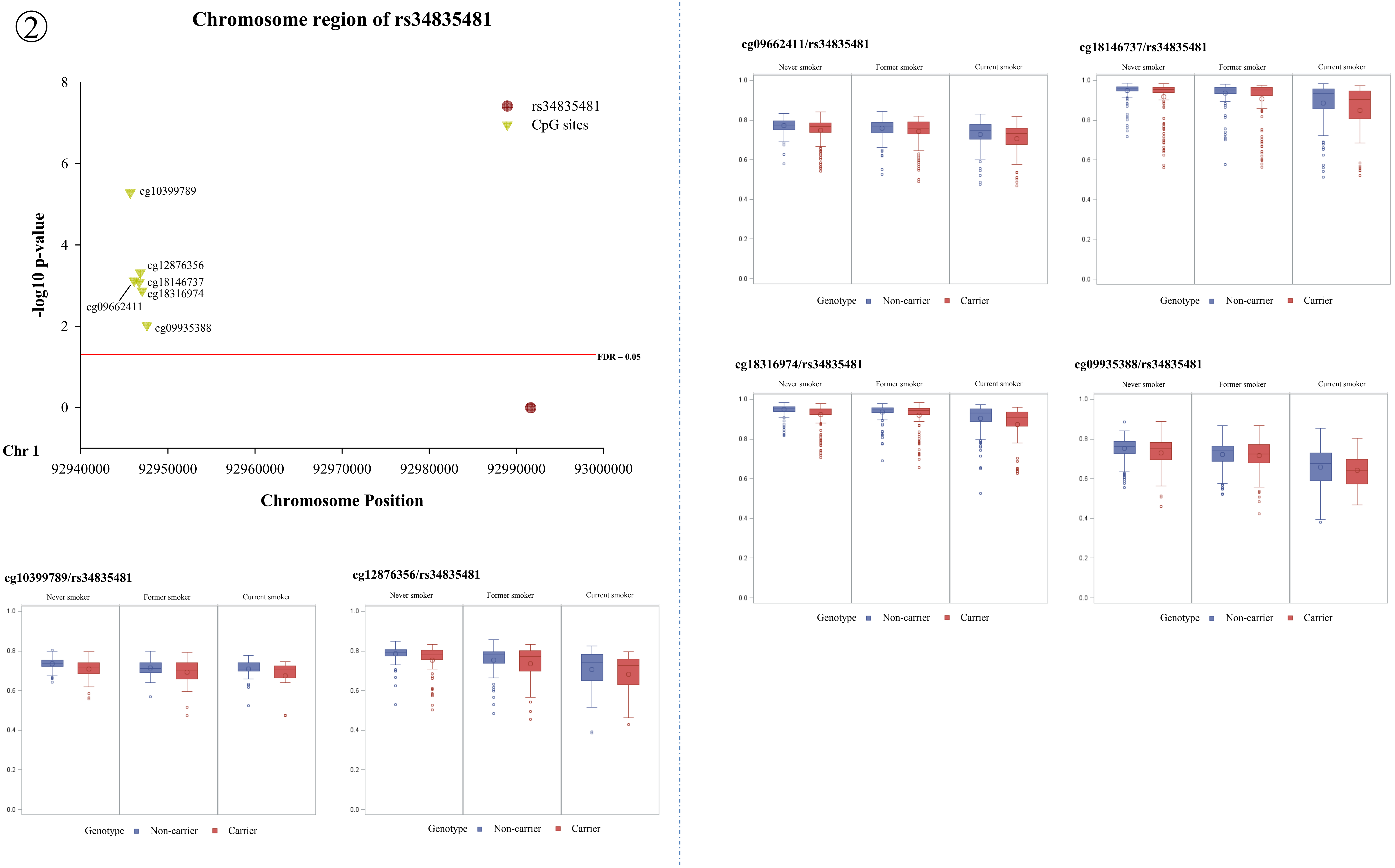
②
Chromosome region of rs34835481
rs34835481
CpG sites
cg10399789
cg12876356
cg18146737
cg18316974
cg09662411
cg09935388
FDR = 0.05
-log10 p-value
Chr 1
92940000
92950000
92960000
92970000
92980000
92990000
93000000
Chromosome Position
cg09662411/rs34835481
cg18146737/rs34835481
cg18316974/rs34835481
cg09935388/rs34835481
cg10399789/rs34835481
cg12876356/rs34835481
Never smoker
Former smoker
Current smoker
Genotype
Non-carrier
Carrier

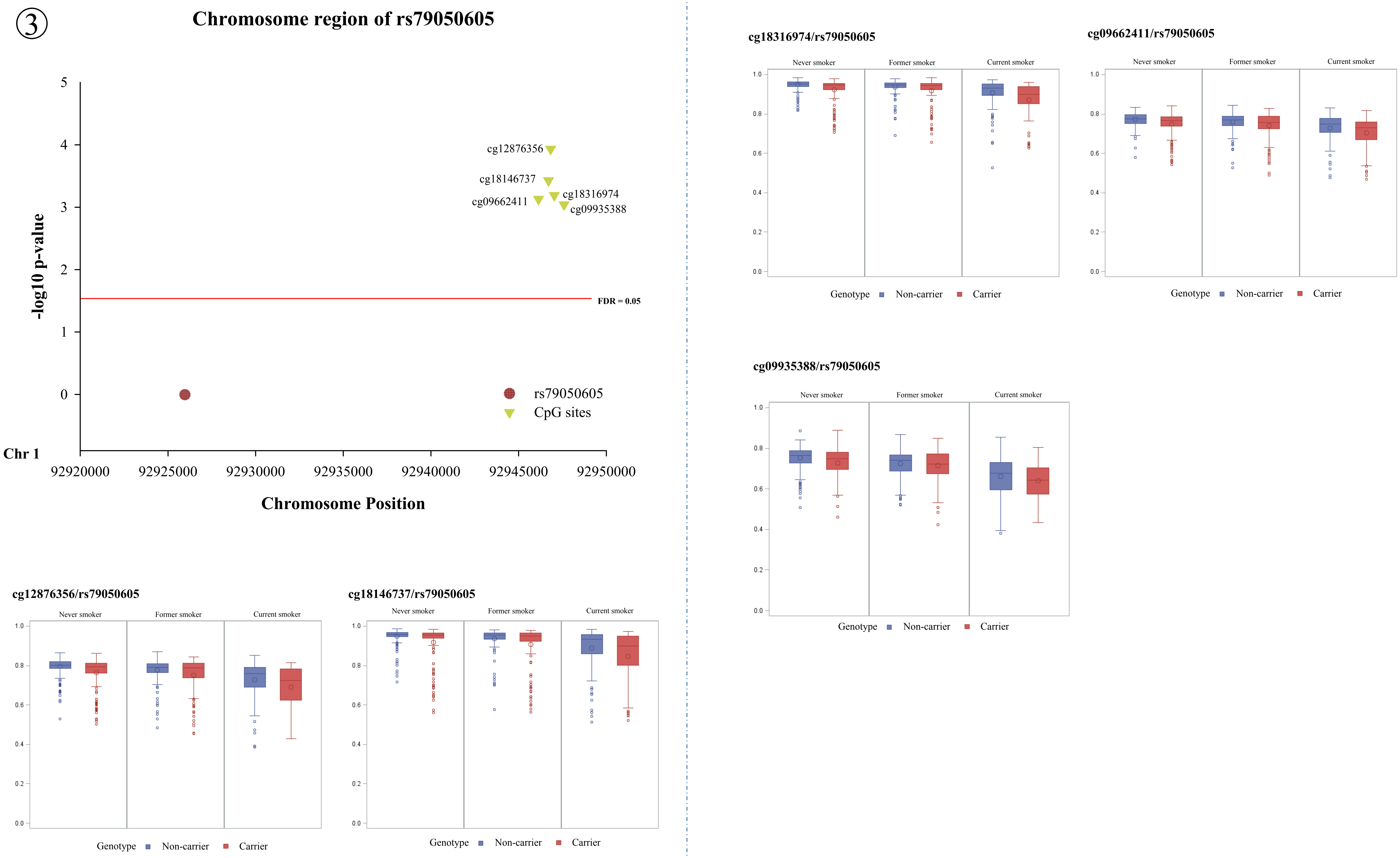
③
Chromosome region of rs79050605
-log10 p-value
5
4
3
2
1
0
cg12876356
cg18146737
cg18316974
cg09662411
cg09935388
FDR = 0.05
rs79050605
CpG sites
Chr 1
92920000
92925000
92930000
92935000
92940000
92945000
92950000
Chromosome Position
cg18316974/rs79050605
cg09662411/rs79050605
cg09935388/rs79050605
cg12876356/rs79050605
cg18146737/rs79050605
Never smoker
Former smoker
Current smoker
1.0
0.8
0.6
0.4
0.2
0.0
Genotype
Non-carrier
Carrier
